# Different dynamics of genome content shuffling among host-specificity groups of the symbiotic actinobacterium *Frankia*

**DOI:** 10.1186/1471-2164-15-609

**Published:** 2014-07-19

**Authors:** Ken-ichi Kucho, Takashi Yamanaka, Hideo Sasakawa, Samira R Mansour, Toshiki Uchiumi

**Affiliations:** Graduate School of Science and Engineering, Kagoshima University, 1-21-35 Korimoto, Kagoshima, 890-0065 Japan; Department of Forest Microbiology, Forestry and Forest Products Research Institute (FFPRI), 1 Matsunosato, Tsukuba, Ibaraki, 305-8687 Japan; Graduate School of Natural Science and Technology, Okayama University, Tsushimanaka, Okayama, 700-8530 Japan; Botany Department, Faculty of Science, Suez Canal University, Ismailia, 41522 Egypt

**Keywords:** Comparative genome hybridization, Nitrogen fixation, Pulsed-field gel electrophoresis, Symbiosis

## Abstract

**Background:**

*Frankia* is a genus of soil actinobacteria forming nitrogen-fixing root-nodule symbiotic relationships with non-leguminous woody plant species, collectively called actinorhizals, from eight dicotyledonous families. *Frankia* strains are classified into four host-specificity groups (HSGs), each of which exhibits a distinct host range. Genome sizes of representative strains of Alnus, Casuarina, and Elaeagnus HSGs are highly diverged and are positively correlated with the size of their host ranges.

**Results:**

The content and size of 12 *Frankia* genomes were investigated by *in silico* comparative genome hybridization and pulsed-field gel electrophoresis, respectively. Data were collected from four query strains of each HSG and compared with those of reference strains possessing completely sequenced genomes. The degree of difference in genome content between query and reference strains varied depending on HSG. Elaeagnus query strains were missing the greatest number (22–32%) of genes compared with the corresponding reference genome; Casuarina query strains lacked the fewest (0–4%), with Alnus query strains intermediate (14–18%). In spite of the remarkable gene loss, genome sizes of Alnus and Elaeagnus query strains were larger than would be expected based on total length of the absent genes. In contrast, Casuarina query strains had smaller genomes than expected.

**Conclusions:**

The positive correlation between genome size and host range held true across all investigated strains, supporting the hypothesis that size and genome content differences are responsible for observed diversity in host plants and host plant biogeography among *Frankia* strains. In addition, our results suggest that different dynamics of shuffling of genome content have contributed to these symbiotic and biogeographic adaptations. Elaeagnus strains, and to a lesser extent Alnus strains, have gained and lost many genes to adapt to a wide range of environments and host plants. Conversely, rather than acquiring new genes, Casuarina strains have discarded genes to reduce genome size, suggesting an evolutionary orientation towards existence as specialist symbionts.

**Electronic supplementary material:**

The online version of this article (doi:10.1186/1471-2164-15-609) contains supplementary material, which is available to authorized users.

## Background

*Frankia* is a genus of soil actinobacteria with unique abilities to fix atmospheric dinitrogen (N_2_) and establish endosymbiotic associations with actinorhizal plants comprising various non-leguminous trees from eight dicotyledonous families [1–3]. This symbiosis, in which *Frankia* reduces N_2_ to ammonium and supplies the resulting product to host plants, takes place in root nodules. As a result of the symbiosis, actinorhizal plants grow rapidly, even in nutrient-poor soils, and improve soil fertility. *Frankia* strains are classified into four host-specificity groups (HSGs) that establish symbiosis with distinct host plant families [4]. “Alnus” strains infect plant species in Myricaceae and the genus *Alnus* of Betulaceae. “Casuarina” strains infect plant species in the genera *Casuarina* and *Allocasuarina* of Casuarinaceae. “Elaeagnus” strains exhibit a broader host range, infecting plant species in five families (including Elaeagnaceae) of the orders Fagales and Rosales. “Rosaceous” strains infect plant species in four families of orders Rosales and Cucurbitales, although no strains have yet been isolated in pure culture. In a phylogenetic tree generated from 16S rDNA sequences, strains belonging to each HSG cluster together in distinct clades [4].

In 2007, complete genome sequences were determined for representative *Frankia* strains from Alnus, Casuarina, and Elaeagnus HSGs [4]. A surprising finding was that despite close phylogenetic relationship (>97.8% identity for 16S rDNA), genome sizes were very different among HSGs. The largest genome (Elaeagnus strain EAN1pec) is 9.0 Mbp and contains approximately 7,400 genes, whereas the smallest one (Casuarina strain CcI3) is only 5.4 Mbp and comprises about 4,600 genes. Alnus strain ACN14a possesses an intermediate-sized genome (7.5 Mbp) of approximately 6,800 genes. This size divergence is the largest reported for any such closely related soil bacteria. Genome size of these strains correlates with the breadth of their host ranges. Comparative genome analysis has revealed that the difference in genome size is due to acquisition, loss, and duplication of genes occurring at different rates in different strains [4].

Two studies have uncovered evidence suggesting how such extensive diversification has occurred in *Frankia* genomes. Since they are particularly prevalent in *Frankia* genomes and indeed retain the ability to be excised from chromosomes, actinomycete integrative and conjugative elements (AICEs) may play a role in gene loss and acquisition [5]. Homologous recombination between insertion sequences (IS) could have also caused deletions of chromosomal segments, as genes contained in IS-rich regions of ACN14a and EAN1pec genomes are absent in the smallest genome, that of CcI3 [6].

In the present study, we analyzed content and size of 12 *Frankia* genomes using *in silico* comparative genome hybridization (CGH) and pulsed-field gel electrophoresis (PFGE) to investigate within-HSG diversity of *Frankia* genomes.

## Results

### Genome sequencing of *Frankia*strains

**Figure 1:**
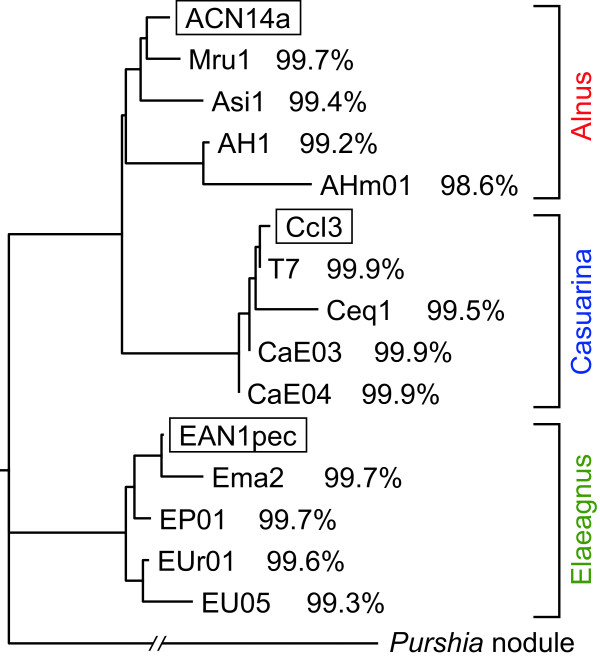
**Phylogenetic tree constructed from 16S rDNA sequences of** ***Frankia*** **strains used in this study.** Reference strains are boxed. Nucleotide sequence identities (%) between query and reference strains of the same host-specificity group (HSG) are shown. Identities between strains of distinct HSGs were 98–99% for Alnus vs. Casuarina HSGs, 97–98% for Casuarina vs. Elaeagnus HSGs, and 97–98% for Alnus vs. Elaeagnus HSGs. *Frankia* from a *Purshia tridentata* nodule was used as an outgroup.

**Figure 2:**
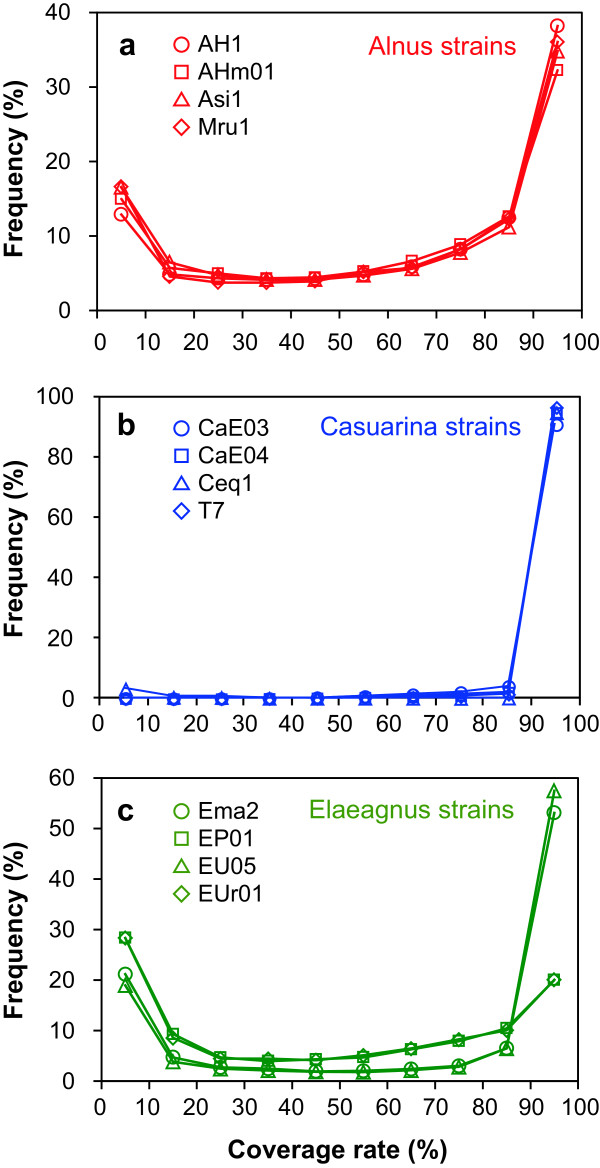
**Histograms of coverage rate for all segments in (a) Alnus, (b) Casuarina, and (c) Elaeagnus strains.** The total number of segments was 12,307 for Alnus, 8,278 for Casuarina, and 13,274 for Elaeagnus strains.

We analyzed genomes of four strains each of Alnus, Casuarina, and Elaeagnus HSGs (Table 1). Strains belonging to the same HSG were phylogenetically very close, showing > 99% identity in 16S rDNA sequences (Figure 1) and > 95% identity in *gyrB* (DNA gyrase subunit B gene) and *recA* (recombinase A gene) sequences (data not shown). We obtained tens of millions of 50-bp reads from each query genome and conducted *in silico* CGH (Additional file 1) using a reference genome from the same HSG: ACN14a for Alnus, CcI3 for Casuarina, and EAN1pec for Elaeagnus HSGs (Table 1). Figure 2 contains histograms of coverage rates for all segments. Distributions were bimodal; most segments displayed either very low (0–10%) or very high (90–100%) coverage rates, with few intermediate values. This result indicates that *in silico* CGH (Additional file 1) can discriminate among genes shared between reference and query genomes and those absent in a query genome. Hereafter, we refer to segments that showed coverage rates of < 20% as low-coverage-rate (LCR) segments, consisting of LCR genes and LCR intergenic regions (IGRs). An LCR segment is likely absent from a query genome, either as a consequence of its deletion from the query genome or its insertion into a reference genome.

**Table 1:** ***Frankia*** **strains used in this study**

HSG	Strain	Source plant	Geographic origin	Usage	No. read
Alnus	ACN14a [7]	*Alnus viridis* subsp. *crispa*	Quebec, Canada	Reference	-
	AH1^a^	*A. hirsuta*	Aomori, Japan	Query	49,569,598
	AHm01^a^	*A. hirsuta* ssp. *microphylla*	Iwate, Japan	Query	35,732,054
	Asi1 [8]	*A. sieboldiana*	Okayama, Japan	Query	48,189,821
	Mru1 [8]	*Myrica rubra*	Okayama, Japan	Query	34,687,733
Casuarina	CcI3 [9]	*Casuarina cunninghamiana*	Petersham, U. S. A.	Reference	-
	CaE03 [10]	*C. equisetifolia*	Okinawa, Japan	Query	46,493,718
	CaE04^a^	*C. equisetifolia*	Senegal	Query	38,361,206
	Ceq1 [8]	*C. equisetifolia*	Okayama, Japan	Query	46,629,137
	T7^a^	*C. cunninghamiana*	Ismailia, Egypt	Query	44,174,301
Elaeagnus	EAN1pec [11]	*Elaeagnus angustifolia*	Ohio, U. S. A.	Reference	-
	Ema2 [8]	*E. macrophylla*	Okayama, Japan	Query	37,558,396
	EP01^a^	*E. pungens*	Kagoshima, Japan	Query	43,449,878
	EU05^a^	*E. umbellata*	Toyama, Japan	Query	26,180,153
	EUr01^a^	*E. umbellata* ssp. *rotundifolia*	Tokyo, Japan	Query	42,309,826

^a^Obtained in this study.

**Figure 3:**
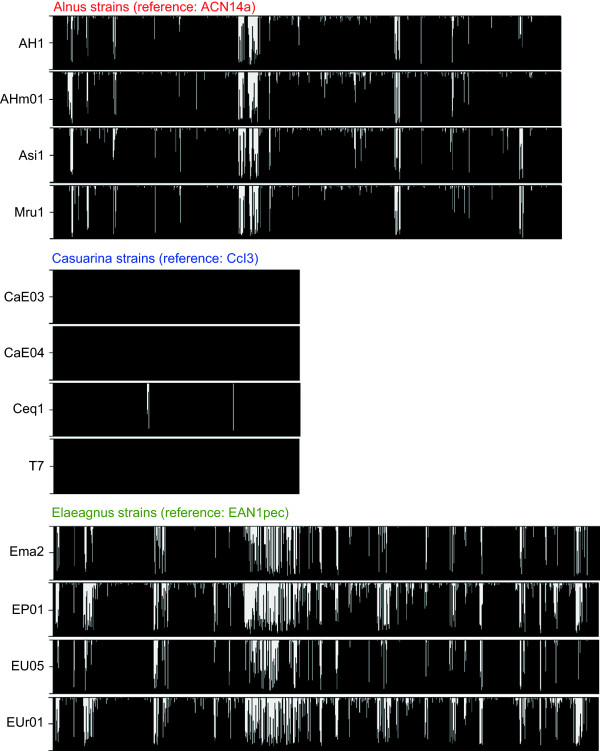
**Distribution of coverage rates over genomes.** Coverage rates of segments (gene and IGR) are represented by vertical black bars arranged in order of their appearance in the genome. Horizontal and vertical axes indicate segment position and coverage rate (%), respectively.

Table 2 lists the number of LCR genes detected for each query genome. LCR genes were most prominent in Elaeagnus strains, accounting for 22–32% of all genes in the corresponding reference genome. The number of LCR genes varied among Elaeagnus strains; more were absent in strains EP01 and EUr01 than in Ema2 and EU05. Compared with Elaeagnus strains, Alnus strains featured fewer LCR genes, which accounted for 14–18% of genes in the reference genome. In Casuarina strains, LCR genes were much rarer; they were not detected for two strains (CaE04 and T7), and accounted for at most only 4% of total genes (Ceq1). We plotted coverage rates of all segments in order of their appearance in the reference genome (Figure 3). LCR segments did not distribute randomly, but tended to be clustered in particular regions of the reference genomes.

**Figure 4:**
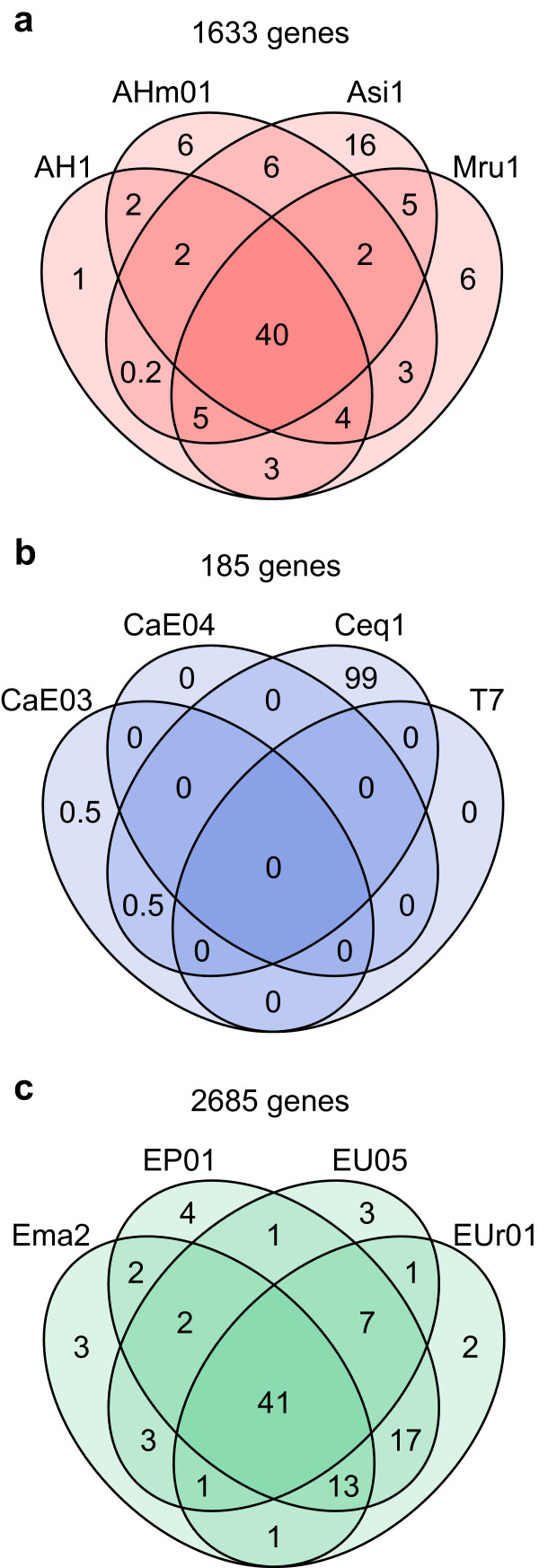
**Venn diagram representing overlap of LCR genes among** ***Frankia*** **strains belonging to (a) Alnus, (b) Casuarina, and (c) Elaeagnus HSGs.** The total number of nonredundant LCR genes is shown above the diagram. Numbers in the diagram are percentages of the nonredundant LCR genes associated with the indicated overlapping strains.

In genomes of ACN14a, CcI3, and EAN1pec, respectively, 1,633, 185, and 2,685 genes were scored as LCR genes for at least one of the four query strains (nonredundant LCR genes; Figure 4). In Alnus and Elaeagnus HSGs, about 40% of nonredundant LCR genes were scored as LCR genes for all four query strains (Figure 4), indicating that they were commonly absent in genomes of these strains. The remaining genes were scored as LCR genes for one to three strains; the distribution of these genes was apparently unbiased, except that LCR genes specific to Asi1 and those shared with EP01 and EUr01 predominated. In the Casuarina HSG, 99% of nonredundant LCR genes were missing only in strain Ceq1; only a few or no LCR genes were associated with the other strains.

**Table 2:** **Number of low-coverage-rate (LCR) genes**

HSG	Reference	No. of genes ^a^	Query	No. of LCR genes	Percentage ^b^ (%)
Alnus	ACN14a	6774	AH1	912	14
			AHm01	1046	15
			Asi1	1241	18
			Mru1	1107	16
Casuarina	CcI3	4569	CaE03	2	0.04
			CaE04	0	0
			Ceq1	184	4
			T7	0	0
Elaeagnus	EAN1pec	7250	Ema2	1743	24
			EP01	2313	32
			EU05	1561	22
			EUr01	2245	31

^a^Total number of protein-coding and RNA genes in the reference genome.

^b^Percentage of genes in the reference genome with low-coverage rates (LCR genes).

### Confirmation by PCR

We used PCR to confirm whether the identified LCR segments were structurally missing in the query genomes. We designed primer sets that flanked individual or clustered LCR segments and performed PCR using Asi1, Ceq1, and Ema2 genomic DNA as templates. Most amplification products (69% for Asi1, 100% for Ceq1, and 75% for Ema2) were smaller than the size expected based on reference genome sequences (Additional file 2), indicating that those LCR segments were missing in the query genomes. In contrast, some bands were larger than the expected size, suggesting insertion of DNA segments at these loci (Additional file 2).

### LCR gene properties

**Figure 5:**
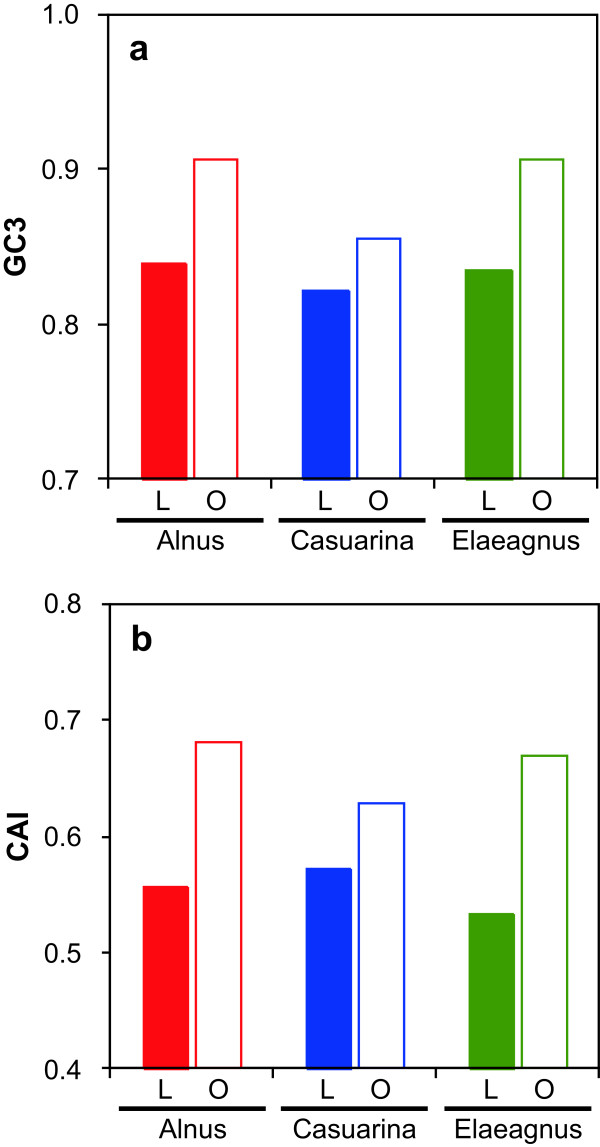
**Average (a) GC content at the third codon position (GC3) and (b) codon adaptation index (CAI) of nonredundant LCR genes (L, solid bar) and other genes (O, open bar).**

GC content at the third codon position (GC3) and codon adaptation index (CAI) of all genes are shown in Figure 5. Nonredundant LCR genes exhibited lower average GC3 and CAI values than other genes, suggestive of foreign origin, possibly through horizontal gene transfer. Dominant functions of nonredundant LCR genes are listed in Table 3. In all HSGs, the vast majority (40–65%) encoded hypothetical proteins with unknown functions. Three functional categories—transcriptional regulation, transport-associated, and transposase—were commonly associated with genes in the three HSGs. In the Alnus HSG, functional categories related to nonribosomal peptide and polyketide synthetases and acyl-CoA metabolism, involved in synthesis of bioactive secondary metabolites such as antibiotics and siderophores [12], were prevalent. In the Casuarina HSG, bacteriophage-related functions, such as restriction and modification system, CRISPR [13], integrase, and excisionase, were prominent.

**Table 3:** **Dominant functions of the top 10 nonredundant LCR genes identified in each studied** ***Frankia*** **host-specificity group**

Function	Percentage
Alnus	
Hypothetical, conserved hypothetical, unknown function	65
Transcriptional regulation	6.0
Transport-associated	3.8
Protein kinase	1.7
Nonribosomal peptide and polyketide synthetases	1.2
Transposase	1.2
Acyl-CoA metabolism	1.0
Amino acids metabolism	0.9
Casuarina	
Hypothetical, conserved hypothetical, unknown function	49
Transposase	9.1
Transcriptional regulation	5.4
Transport-associated	4.3
Restriction and modification system	2.2
CRISPR associated	2.2
Integrase	1.6
Putative ATP/GTP-binding protein	1.6
AMP-dependent synthetase and ligase	1.6
DNA synthesis	1.1
NUDIX hydrolase	1.1
Excisionase	1.1
Elaeagnus	
Hypothetical, conserved hypothetical, unknown function	40
Transcriptional regulation	7.9
Transposase	6.0
Transport-associated	5.3
Short-chain dehydrogenase/reductase	2.8
Acyl-CoA metabolism	1.8
Protein kinase	1.3
Methyltransferase of unknown function	1.2
Integrase	1.1
Alcohol/Aldehyde dehydrogenase	1.0

### PFGE

**Figure 6:**
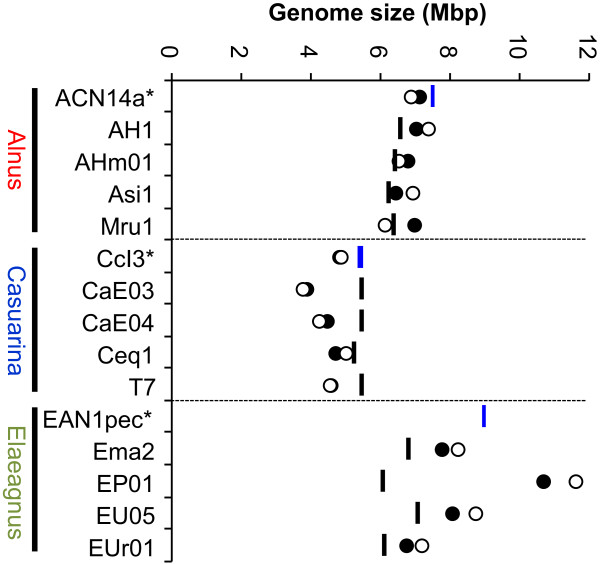
**Genome size estimated by PFGE.** Sizes estimated with *Dra*I- and *Psi*I-digested genomic DNAs are indicated by closed and open circles, respectively. Blue bars represent actual genome sizes of reference strains (indicated by asterisks). Black bars correspond to expected sizes of query genomes, calculated by subtracting total length of LCR segments from reference genome size.

We estimated genome sizes of studied *Frankia* strains via PFGE of genomic DNA digested with *Dra*I or *Psi*I. Sizes obtained using either restriction enzyme were mostly consistent (Figure 6). Results from two reference strains (ACN14a and CcI3) revealed that the estimated sizes were slightly smaller than actual genome sizes (Figure 6) for two reasons: i) small bands less than 50 kb migrated out of the gel; and ii) the relative migration rate of *Frankia* DNA was faster than that of yeast marker DNA (Additional file 3). Expected sizes of query genomes, based on the assumption that they lacked all LCR segments, are shown in Figure 6. Genome sizes of the four Alnus query strains after the above underestimation was taken into account were larger than expected (Figure 6), but were similar to that of the reference genome (ACN14a). Estimated genome sizes of the four Elaeagnus query strains were apparently larger than expected. Two strains (Ema2 and EU05) appeared to have genome sizes similar to the reference strain EAN1pec when underestimation was taken into account. Notably, the estimated genome size of EP01, in spite of the absence of more than 30% of genes, was much larger than that of EAN1pec (Table 2). An opposite situation was observed in Casuarina query strains. Although few genes were missing in genomes of CaE03, CaE04, and T7 (Table 2), their estimated genome sizes were significantly smaller than the reference strain CcI3. Little similarity in banding patterns was observed among or even within HSGs (Additional file 3), suggesting divergence of genome structure. As reported for the reference strains [4], genome sizes of the query strains were correlated with extent of their host ranges: Casuarina strains possessed the smallest genomes, Elaeagnus strains the largest, with Alnus strains intermediate.

### Clustering of LCR genes

**Figure 7:**
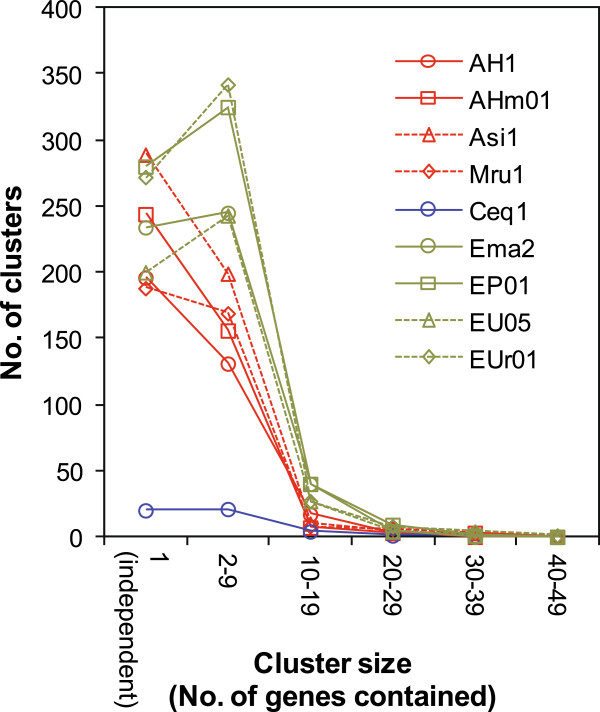
**Size and number of LCR gene clusters.** The number of LCR gene clusters containing the indicated number of genes, as well as the number of independent LCR genes, is shown for each studied query strain.

To evaluate clustering of LCR genes, we searched reference genomes for consecutive arrays of LCR segments, which were considered to be LCR gene clusters if they contained two or more LCR genes. Size and number of identified LCR gene clusters are shown in Figure 7. In every HSG, the vast majority of clusters were small, containing less than 10 genes. A substantial number of independent LCR genes that did not form clusters were also detected (Figure 7). Fewer LCR gene clusters were found in Ceq1 since the strain is associated with only a small number of LCR genes (Table 2). Data for strains CaE03, CaE04, and T7 was not shown because they were associated with a few or no LCR genes (Table 2) and no LCR gene cluster was detected.

LCR gene clusters would be a kind of genomic islands typically integrated into chromosomes by site-specific recombination at a tRNA gene through the action of integrase [5, 14]. We searched both ends of each LCR gene cluster for direct repeats of tRNA sequences, but only two clusters were associated with such sequences (CcI3: Francci3_1194 to IGR Francci3_1203-Francci3_R0023, tRNA-Gly; EAN1pec: IGR Franean1_R0059-Franean1_7129 to Franean1_7139, tRNA-Glu) (Table 4).

**Table 4:** **LCR gene clusters associated with potential insertion/deletion elements**

Reference genome	Total ^a^	tRNA repeats	Direct repeats ^b^	Same ISs
ACN14a	578	0	17	0
CcI3	27	1	0	0
EAN1pec	1090	1	35	8

^a^Number of nonredundant LCR gene clusters found in all query strains.

^b^Direct repeats of tRNA sequences are not included.

AICEs are prevalent in *Frankia* genomes, and have been experimentally confirmed to retain their ability to be excised from chromosomes [5]. Although three AICEs have been identified in ACN14a and CcI3 genomes, and four in the EAN1pec genome [5], only one AICE—in ACN14a (Faln2929)—corresponded closely to any LCR gene clusters in our study (data not shown).

DNA segments flanked by homologous sequences (direct repeat sequences) or flanked by ISs from the same family can be excised from chromosomes. LCR gene clusters associated with direct repeat sequences were relatively frequent in ACN14a and EAN1pec, but nevertheless represented only a small fraction of observed clusters (Table 4). In the EAN1pec genome, several clusters were associated with ISs belonging to the same family. To summarize, however, few LCR gene clusters were associated with elements previously known to be involved in insertion and deletion of DNA segments.

## Discussion

In this study, we used an *in silico* CGH method based on the application of next-generation sequencing technology. Resolution obtained using *in silico* CGH is higher than that available from DNA array-based CGH. In addition, *in silico* CGH experiments are less time-consuming, as this technique does not require construction of DNA arrays. On the other hand, this method is inferior to comparative analyses that use assembled genome sequences and homology search programs. When a large number of undetermined genomes need to be compared, however, *in silico* CGH is useful, because complete genome assembly is very laborious.

Recent comparative genomic studies have revealed that bacterial genome contents vary greatly, even among closely related species and strains [15–19]. As confirmed in our study, this is also true for Alnus and Elaeagnus HSG *Frankia* strains. An unexpected and novel finding of our study, however, is that this diversity varies depending on the HSG. Alnus strains lacked 14–18% of genes present in a reference genome from the same HSG, whereas more than 20% of genes were absent in Elaeagnus strains, with over 30% lacking in two strains (EP01 and EUr01) (Table 2). These divergences are much greater than that observed between the actinobacterial species *Streptomyces coelicolor* and *Streptomyces lividans* (7%) [15]. In the case of *Escherichia coli*, a comparable level (about 25%) of divergence occurs between pathogenic and non-pathogenic strains [16]. Because *Frankia* and *Streptomyces* occupy the same ecological niche, i.e. soil, environmental factors offering differing opportunities for horizontal gene exchange within the bacterial community cannot be responsible for the discrepancy. Inherent properties specific to *Frankia*, such as domino effects (see below), may allow such dynamic changes.

In spite of these remarkable levels of gene loss, PFGE revealed that actual genome sizes of Alnus and Elaeagnus query strains were not as small as expected based on total LCR gene length (Figure 6). This result indicates that these query strains carry genes that are absent in the reference genomes, thus compensating for the reduced genome size due to gene loss. Insertion of additional DNA segments was indeed observed in query strains (Additional file 2) based on genomic PCR. These strains have thus both lost and acquired significant numbers of genes over the course of evolution; as a consequence, genome contents have diverged greatly, even within the same HSG. Interestingly, such shuffling of genome content appears to have occurred to different extents between the two HSGs. More dynamic shuffling has taken place in genomes of Elaeagnus strains than in Alnus, as evidenced by the greater extent of gene loss (number of LCR genes; Table 2) and higher compensated genome size (Figure 6) in the Elaeagnus HSG. In Alnus strains, gene acquisition and loss seems to have been mostly balanced, because the number of LCR genes and genome size are similar among strains (Table 2 and Figure 6); this balance was not well maintained in Elaeagnus strains.

Unlike the other two HSGs, very few LCR genes were identified among Casuarina strains (Table 2), indicating that genome contents were highly similar within the HSG. In particular, strains CaE03 and CaE04 were missing only two or no LCR genes, respectively (Table 2), revealing that these query strains possessed almost all the genes in the reference genome (CcI3). Genome sizes of the query strains were significantly smaller than that of CcI3, however (Figure 6). These results suggest that some components of multigene families in the CcI3 genome were missing in CaE03 and CaE04. Normand et al. [4] have pointed out that transposase genes are frequently duplicated in the CcI3 genome, forming large multigene families. Loss of such transposase genes may consequently be responsible for the observed size reductions.

When complete sequences were obtained for three representative *Frankia* strains, the most surprising finding was their unusual size divergence. To explain the biological significance of this divergence, genome size and content have been proposed to influence host range and biogeographical adaptation of bacterial strains [4]. Casuarina strain CcI3 has the smallest genome, consistent with the narrowest range of hosts and the limited environment of its host plants’ habitat (temperate regions of Australia) [20]. In contrast, Elaeagnus strain EAN1pec has the largest genome, helping it to achieve the broadest host range and to adapt to the wide range of soil types and climates under which its host plants grow [20]. Our PFGE results support this hypothesis, as this correspondence between genome size and HSG held true for the 12 strains analyzed (Figure 6). In addition, our results suggest that the dynamics of genome content shuffling, along with genome size, have contributed to these symbiotic and biogeographic adaptations. Genomes of Elaeagnus strains have likely discarded and acquired a greater number of genes to manage adaptation to a wider range of hosts (spanning five families) and encountered environments. Alnus strains may have also done so, but to a lesser extent, because their host range (spanning two families) is not as broad as that of Elaeagnus strains. Indeed, LCR genes are associated with regulatory, metabolic, and transport functions (Table 3) suggestive of such adaptive roles. In *Bradyrhizobium*, acquisition of genomic islands is reported to influence symbiotic nitrogen fixation properties [19]. In contrast to Elaeagnus and Alnus, Casuarina strains have not acquired new genes, but have instead discarded them to reduce their genome sizes; this suggests an evolutionary orientation towards existence as specialist symbionts [4]. Casuarina strains infect only a narrow spectrum of hosts, spanning two genera, and show reduced saprophytic activity which is evidenced by the fact that these strains have not been isolated from soils outside the native habitats of their host plants [21, 22]. Such reductive genome evolution is often observed in obligate symbiotic bacteria [23, 24].

Most detected LCR gene clusters were not flanked by elements known to be associated with genomic islands [14] (Table 4). This is similar to the case of *E. coli* [25]. Because current cluster structure is a product of multiple DNA rearrangement steps, elements functional in the past may no longer be located at cluster termini. We therefore cannot determine whether the disparate occurrence of such elements explains differences in genome stability.

On the other hand, den Bakker et al. [26] have proposed a “domino” effect theory to explain why a particular genomic region is subject to active gene acquisition and loss. If a genome has acquired a genomic island that encodes beneficial gene products, the island will be maintained. Most parts of the island will be functionally neutral, however; they may easily accept insertion and deletion of genes without losing the island’s adaptive value, making the region a hot spot for gene exchange. We can use this hypothesis to explain the different dynamics of genome content shuffling observed in *Frankia*; the more genomic islands (LCR gene clusters) in a genome, the more chances for gene acquisitions and losses.

## Conclusions

Our results suggest that two genomic properties have affected diversity in host plant range and biogeography in *Frankia* strains. The first property, genome size, was previously proposed by Normand et al. [4] and has been validated by our study. The second property is the dynamics of genome content shuffling. In other words, Elaeagnus strains have both retained and exchanged a large number of accessory genes to adapt to diverse host plant species, soil types, and climates. In contrast, Casuarina strains have discarded rather than acquired genes to limit hosts and inhabited environments, suggestive of an evolutionary preference for specialist symbiosis. Differences in the extent of genome content shuffling can be partially explained by domino effects: if a strain carries more genomic islands, then more neutral regions accompany them, thus enhancing genome flexibility towards gene acquisition and loss.

## Methods

### Bacterial strains

*Frankia* strains AH1, AHm01, CaE04, EP01, EU05, and EUr01 were isolated using the differential filtration method [27] from root nodules collected in the field (Table 1). Lobes of fresh nodules were sterilized with 1% sodium hypochlorite for 5 min, washed with sterilized water, and homogenized in a mortar. The homogenates were passed through filters with 50- and 20-μm nylon mesh screens. Plant residues and *Frankia* vesicle clusters collected through filtration were mixed in 100-ml flasks with 40 ml of modified BAP medium [28] lacking ammonium chloride (BAP-). The flasks were placed at 29°C in darkness. *Frankia* strain T7 was isolated from root nodules of *Casuarina cunninghamiana*. The fresh nodules were washed and dissected into individual lobes and surface-sterilized as described in [29]. Each lobe was checked for sterility in sterile nutrient-rich medium. Nodules free from contaminant were dissected and transferred to 125-ml flasks containing modified BAP medium [30] and incubated at 28°C. *Frankia* filaments were homogenized and diluted 1:100 (v/v) with sterile distilled water; 1 ml of the suspension was then transferred to melted agar DPM medium [31]. The plate was agitated, allowed to solidify, and incubated at 28°C for 3 weeks. A single colony was picked up, homogenized, and cultured in liquid B medium [28].

*Frankia* strains were maintained in BAP (ACN14a), BAP- (AH1, AHm01, CaE03, CaE04, T7, EP01, EU05, and EUr01), BAP-T [32] (CcI3), or Qmod [33] (Asi1, Mru1, Ceq1, and Ema2) media in tissue culture flasks (TPP, Trasadingen, Switzerland) at 28°C.

### Genomic DNA preparation

*Frankia* cells were suspended in TE buffer (10 mM Tris-HCl [pH 8.0] and 1 mM EDTA) containing 8 mg ml^-1^ lysozyme, and incubated at 37°C for 1 h. Cells were collected by centrifugation, and genomic DNA was purified using a DNeasy Plant Mini kit (Qiagen, Hilden, Germany) according to the manufacturer’s instructions.

### Phylogenetic analysis of 16S rDNA

The full-length 16S rDNA region was amplified by PCR using primers CcI3 16S rRNA f1 (5'-TTGATGGAGAGTTTGATCCTGG-3') and CcI3 16S rRNA r1 (5'-AGAAAGGAGGTGATCCAGC-3'). Residual primers and nucleotides were removed by exonuclease I (Takara Bio, Ohtsu, Japan) and shrimp alkaline phosphatase (Roche, Mannheim, Germany), and PCR products were directly sequenced using BigDye terminator v3.1 (Applied Biosystems, Foster City, CA, USA). A phylogenetic tree was constructed by the neighbor-joining method [34] using Genetyx (Genetyx, Tokyo, Japan). GenBank accession numbers of generated sequences are as follows: ACN14a, NC_008278.1; AH1, AB849940; AHm01, AB849941; Asi1, AB847121; Mru1, AB848357; CcI3, NC_007777.1; CaE03, AB849939; CaE04, AB849942; Ceq1, AB848358; T7, AB850642; EAN1pec, NC_009921.1; Ema2, AB848359; EP01, AB849943; EU05, AB849944; EUr01, AB849945; and *Purshia* nodule, AF034776.

### Next-generation genomic sequencing

We sequenced genomes of *Frankia* strains using a SOLiD 4 next-generation sequencing system (Applied Biosystems). Libraries were generated from 1 μg genomic DNA using a SOLiD fragment library construction kit. Templated beads were prepared with a SOLiD ePCR kit v2 and XD beads enrichment kit, and then deposited on a glass slide using a SOLiD XD slide and deposition kit v2. Fifty base pairs at the ends of library fragments were sequenced using a SOLiD ToP fragment BC sequencing kit and a SOLiD ToP instrument buffer kit. All experiments were performed according to the manufacturer’s instructions.

### Data analyses

To compare the content of *Frankia* genomes, we used a strategy named *in silico* CGH (Additional file 1), which has been used to find missing genes in bacterial genomes [35]. The 50-bp reads (query reads) obtained from *Frankia* genomes of unknown sequence (query genomes) were mapped to a reference genome whose complete sequence had already been reported [4] — *Frankia* strains ACN14a (GenBank: NC_008278.1), CcI3 (NC_007777.1), and EAN1pec (NC_009921.1). Mapping was conducted using Bioscope software (Applied Biosystems). The term “map” means to match an individual 50-bp query read to a region with significant sequence similarity on a reference genome. Regions of a reference genome onto which few or no reads are mapped were deduced to be absent in the query genome. To quantitatively evaluate the mapping results, we dissected reference genome sequences into two types of segments—gene and IGR—and calculated “coverage rate” of each segment. Coverage rate was calculated as the percentage of nucleotides in the segment that were mapped by one or more reads (Additional file 1). A low coverage rate indicated that few query reads were mapped to a segment in a reference genome; in such cases, that segment was likely absent in the query genome. Coverage rates of all the genes in Alnus, Casuarina and Elaeagnus strains are shown in Additional file 4.

Data for GC3 and CAI were obtained from the MaGe database (https://www.genoscope.cns.fr/agc/mage/). A list of ISs annotated for *Frankia* genomes was taken from Bickhart et al. [6]. We used the ssearch program (http://fasta.bioch.virginia.edu/fasta_www2/fasta_intro.shtml), which implements the Smith-Waterman algorithm [36], to find repeat sequences at ends of LCR gene clusters.

### PCR

We conducted PCR using genomic DNAs (10 ng) of strains Asi1, Ceq1, and Ema2 as templates, along with the primers listed in Additional file 5, GC buffer I (Takara Bio), and *EX Taq* polymerase (Takara Bio).

### Pulsed-field gel electrophoresis

Cells of *Frankia* were harvested from 5–15 ml of culture solution and resuspended in 0.3 ml HE buffer (10 mM 4-[2-hydroxyethyl]-1-piperazineethanesulfonic acid [pH 8.0] and 1 mM EDTA). The cell suspension was mixed with an equal volume of 2% low-melting agarose (Agarose-LM plaque; Nacalai Tesque, Kyoto, Japan) in HE buffer and solidified in plug molds (Bio-Rad, Hercules, CA, USA). The agarose plugs were incubated with 2 mg ml^-1^ lysozyme in HE buffer at 37°C for 2 h, and then with 1 mg ml^-1^ proteinase K (Nacalai Tesque) in NDS buffer (0.5 M EDTA, 10 mM Tris-HCl [pH 8.0], and 1% SDS) at 50°C for 24 h. We removed the proteinase K solution and washed the plugs once with 10 ml HE buffer containing 0.1 mM phenylmethylsulfonyl fluoride (Nacalai Tesque) and three times with HE buffer. The plugs were then washed three times with TE buffer and equilibrated with 1× buffer supplied with the restriction enzymes. We digested DNA in 200 μl solution containing 1× restriction enzyme buffer, 0.5 mM dithiothreitol, 1 mg ml^-1^ bovine serum albumin, and 30 units of *Dra*I (Roche) or 1 μl of FastDigest *Psi*I (Thermo Scientific, Waltham, MA, USA) at 37°C for 5 h or overnight. Electrophoresis was performed under the conditions described in Additional file 3 using the CHEF-DR III system (Bio-Rad). Chromosomes of *Saccharomyces cerevisiae* and *Schizosaccharomyces pombe* were used as size standards (Bio-Rad). Gels were stained with SYBR Gold (Life Technologies, Carlsbad, CA, USA), and electropherograms were obtained under UV irradiation.

### Availability of supporting data

All the data supporting the results of this article are included as additional files.

## Author’s information

Hideo Sasakawa as emeritus professor.

## Electronic supplementary material

Additional file 1: **Schematic overview of** ***in silico*** **CGH.** PDF file (.pdf) explaining *in silico* CGH procedure. (PDF 104 KB)

Additional file 2: **Results of genomic PCR.** PDF file (.pdf) containing electropherograms of PCR products. Genomic DNAs from Asi1, Ceq1, and Ema2 were used as templates. (PDF 208 KB)

Additional file 3: **Electropherograms from PFGE.** PDF file (.pdf) containing gel images from PFGE. Numbers on the left side of the image are Mbp of size standards (chromosomal DNA of *S. cerevisiae* and *S. pombe*). Numbers with arrowheads on the right side of images are fragment sizes estimated from the size standards. Conditions for electrophoresis are indicated under the image: (A) 0.5× TBE (45 mM Tris base, 45 mM borate, and 1 mM EDTA [pH 8.5]) with 10 mM thiourea, 1% agarose, 6 V cm^-1^ voltage, 60–120-s pulse time, 120° field angle, and 24-h run time at 13°C; (B) 1× TAE (40 mM Tris-acetate, 2 mM EDTA [pH 8.5]) with 10 mM thiourea, 0.8% agarose, 2 V cm^-1^ voltage, 1200–1800-s pulse time, 106° field angle, and 48-h run time at 14°C; (C) 1× TAE with 10 mM thiourea, 0.8% agarose, 3 V cm^-1^ voltage, 120–1200-s pulse time, 106° field angle, and 24-h run time at 13°C. (PDF 509 KB)

Additional file 4: **Coverage rates of genes.** Microsoft Excel file showing coverage rates of all genes in Alnus, Casuarina and Elaeagnus strains analyzed in this study. (XLSX 2 MB)

Additional file 5: **List of primers.** Microsoft Excel file containing a list of primers used for genomic PCRs of Additional file 2. (ODS 18 KB)
