# Supplementary figures and images for: Different dynamics of genome content shuffling among host-specificity groups of the symbiotic actinobacterium Frankia
Source: BMC Genomics. 2014 Jul 19;15(1):609. doi: 10.1186/1471-2164-15-609 (PMC4117964; doi:10.1186/1471-2164-15-609)

# Additional file 1

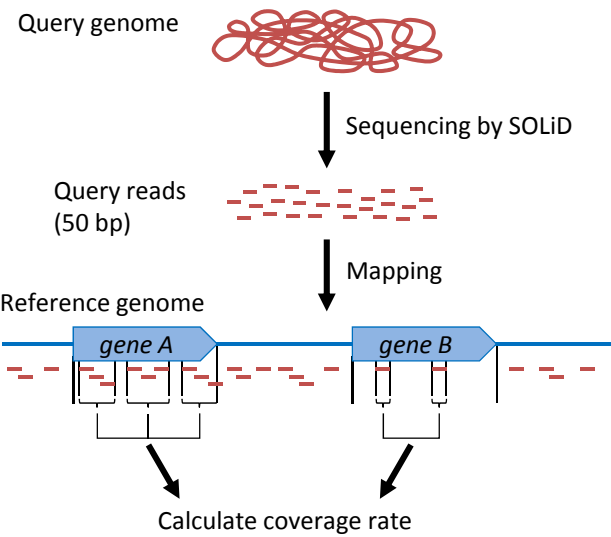

Supplement: Supplementary file 1 — Additional file 1: Schematic overview of in silico CGH. PDF file (.pdf) explaining in silico CGH procedure. (PDF 104 KB) [file 12864_2013_6303_MOESM1_ESM.pdf]

Additional file 3

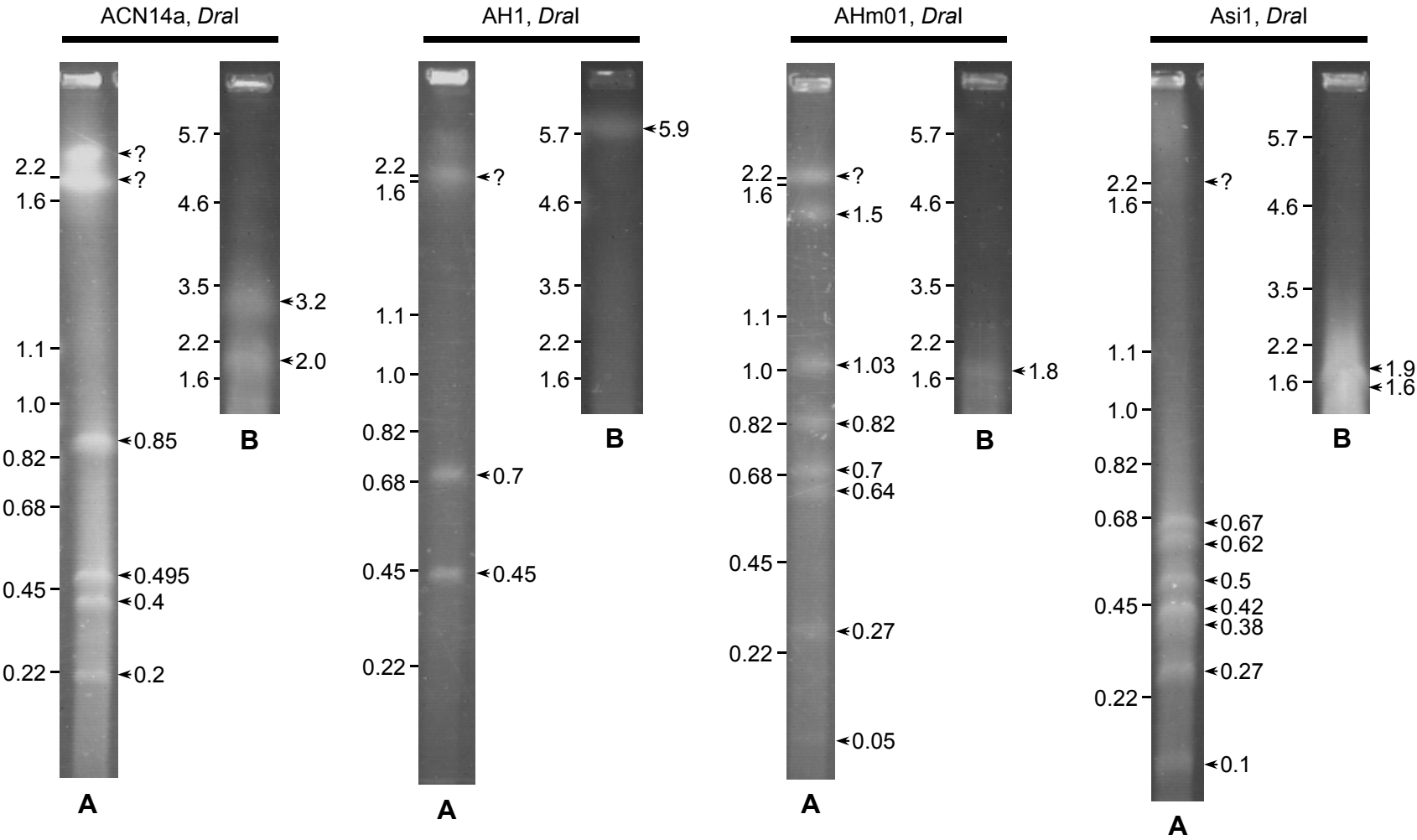

Mru1, *DraI*

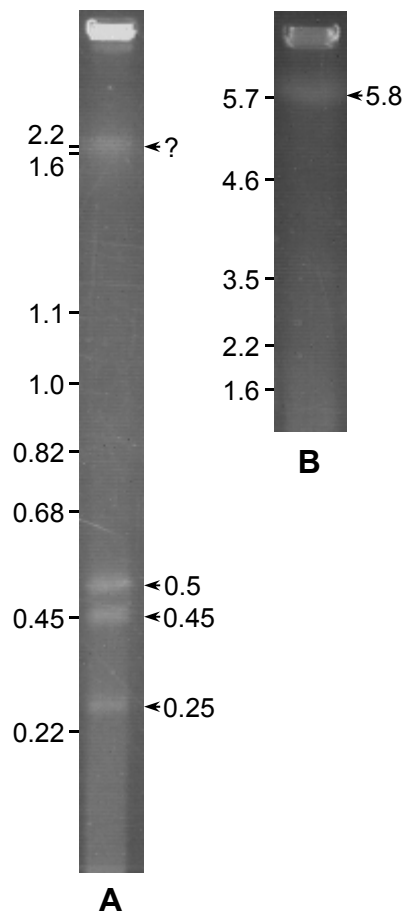

ACN14a, *PsiI*

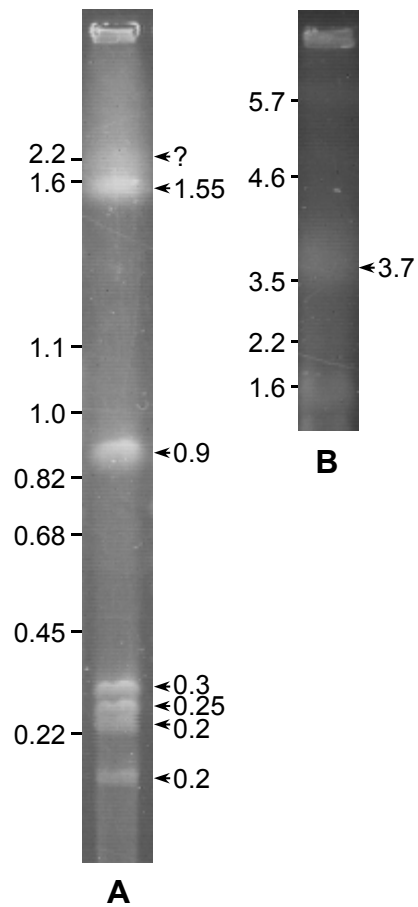

AH1, *PsiI*

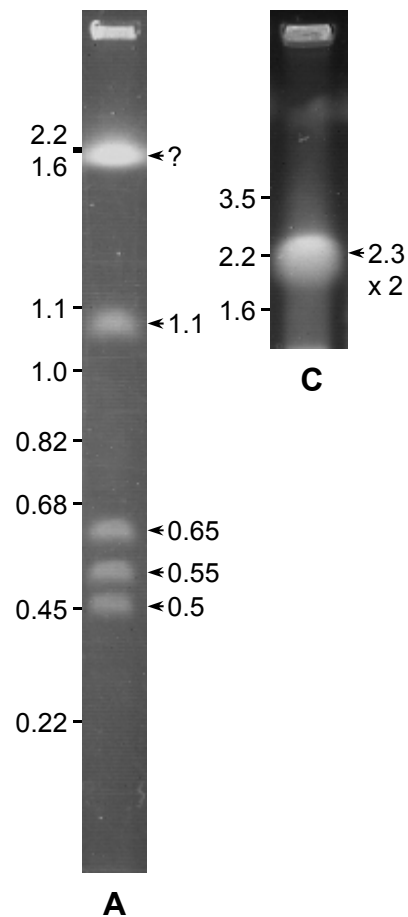

AHm01, *PsiI*

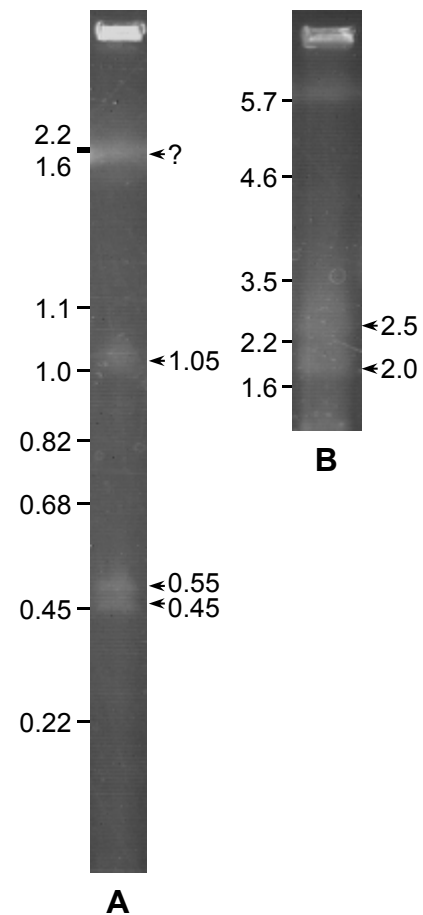

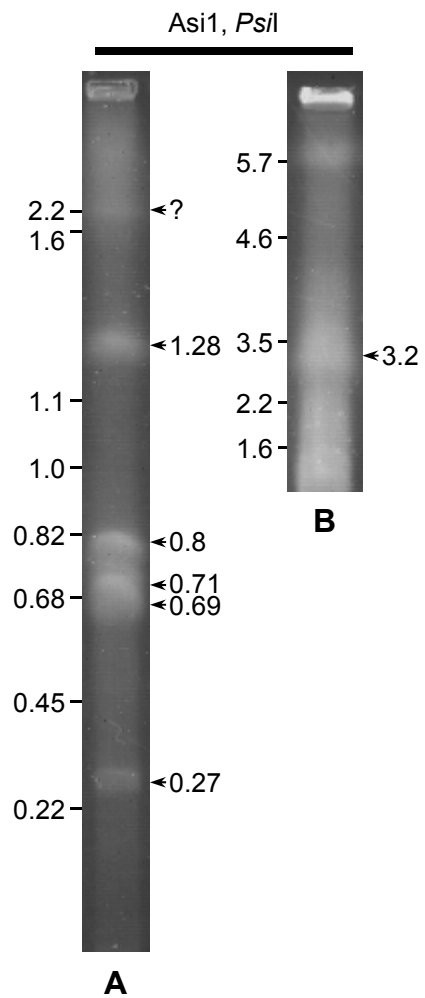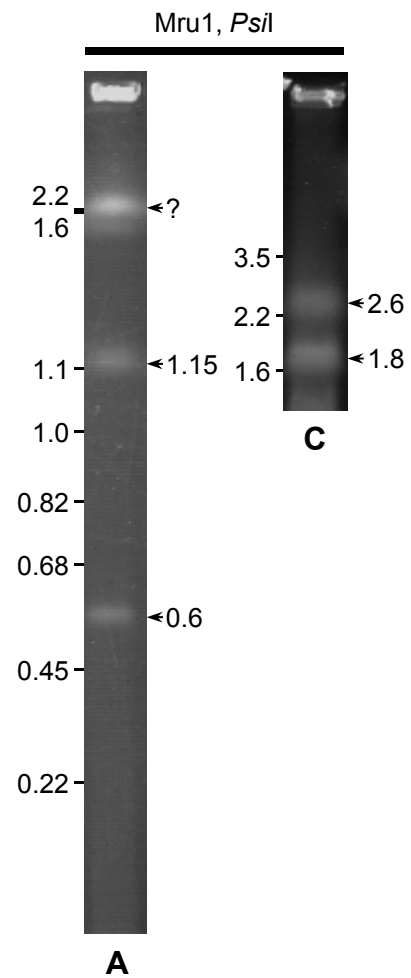

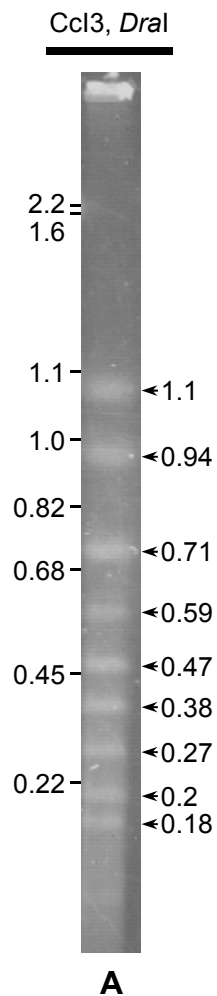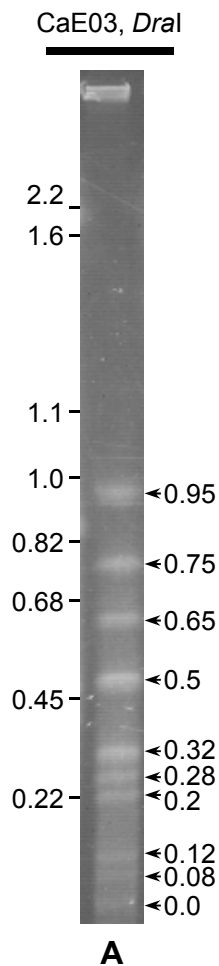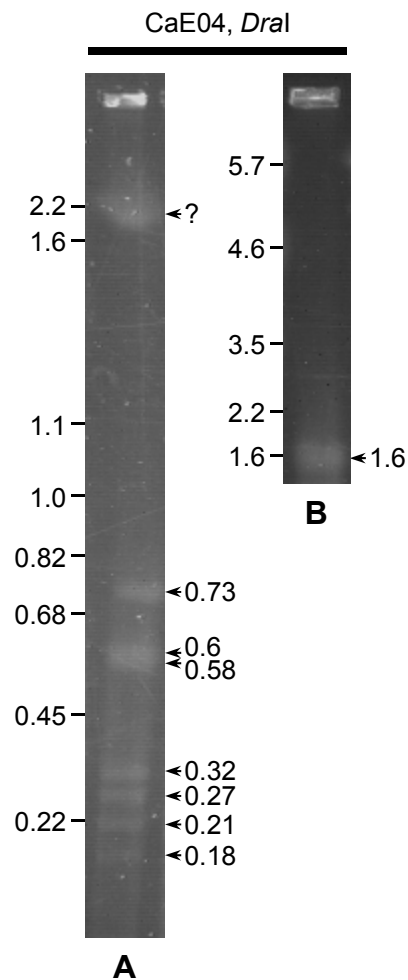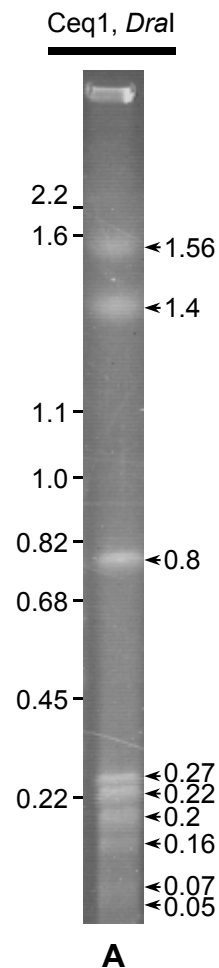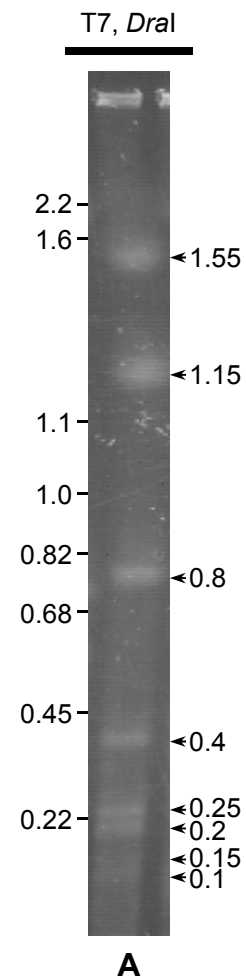

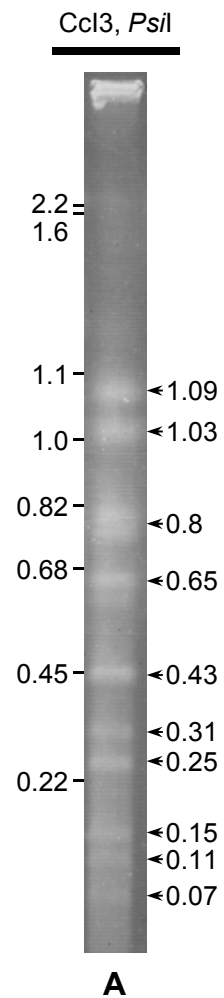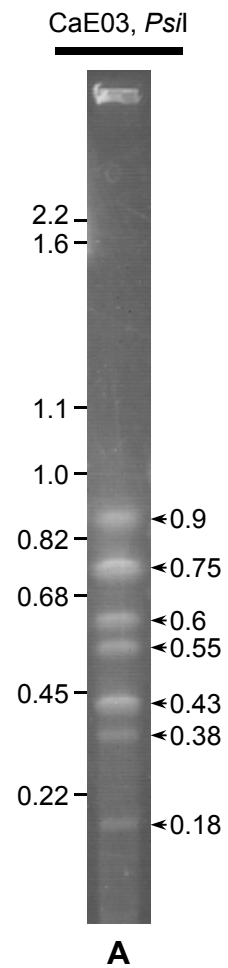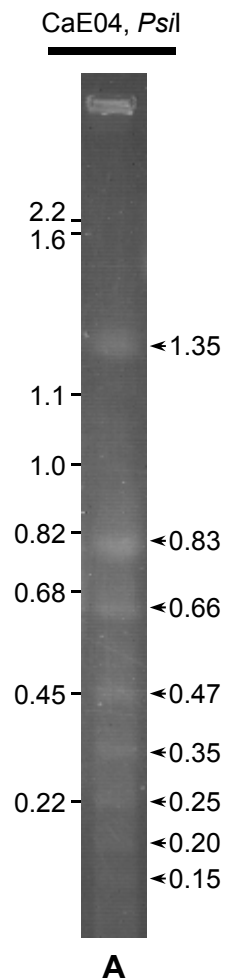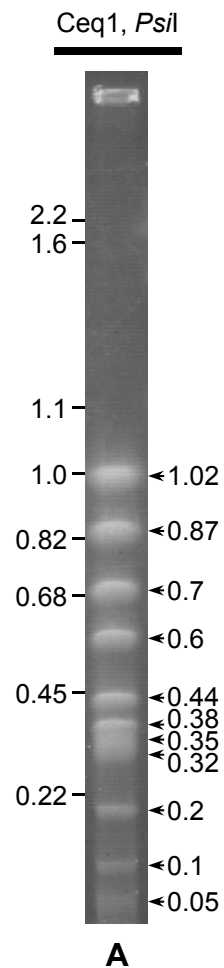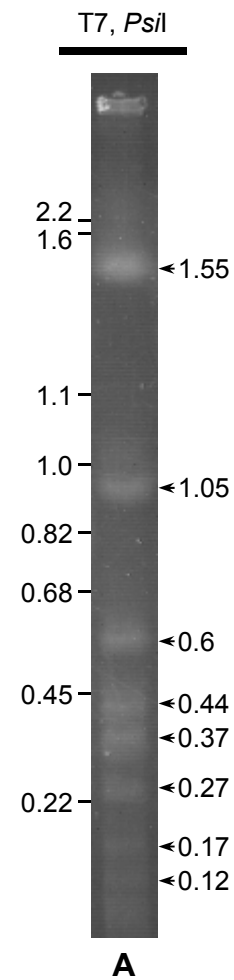

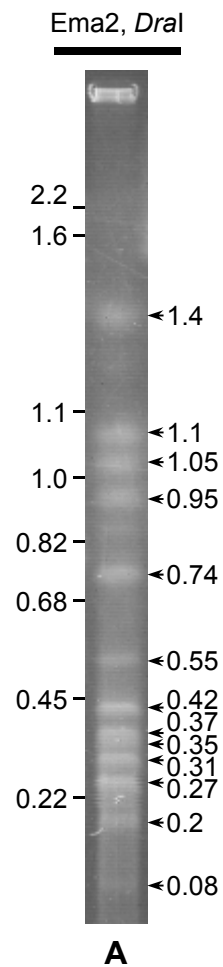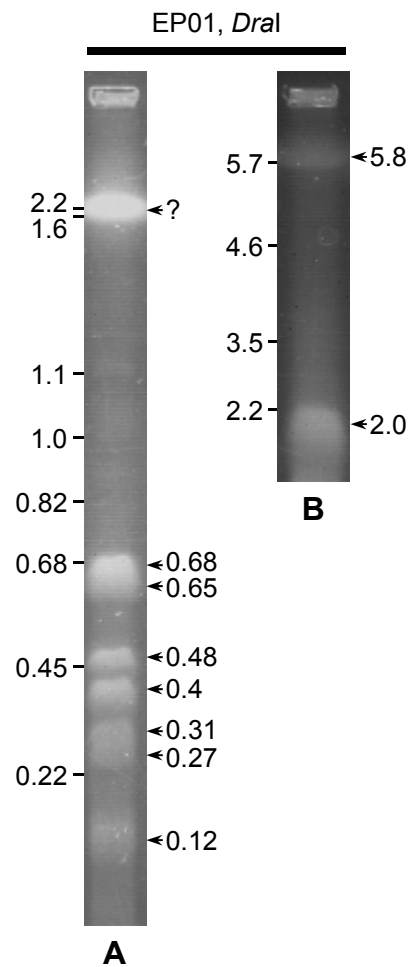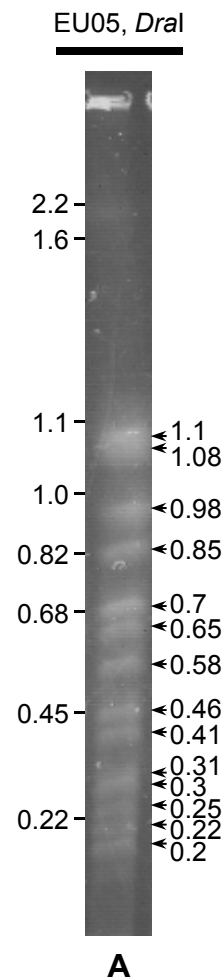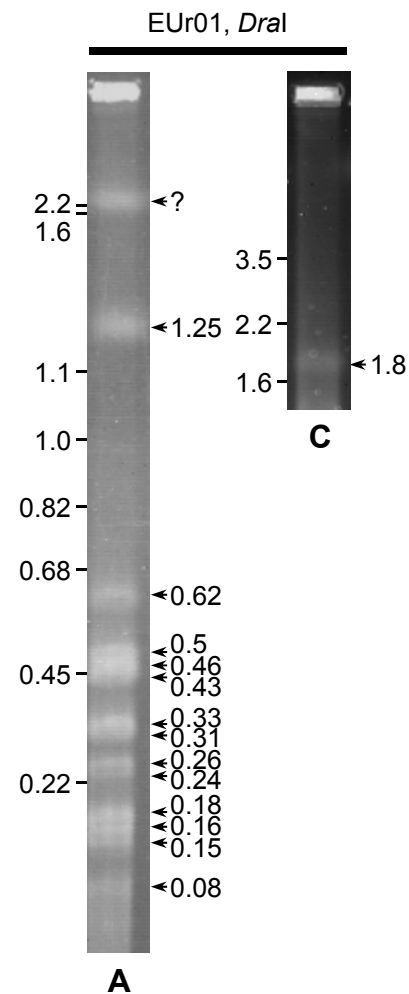

Ema2, *PsiI*

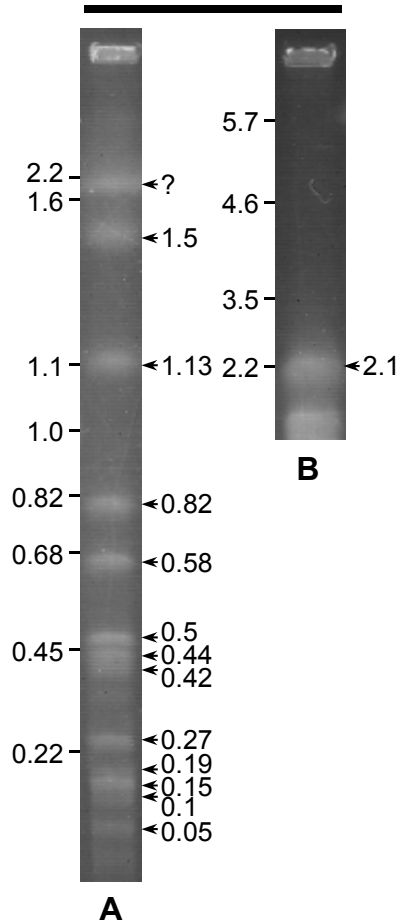

EP01, *PsiI*

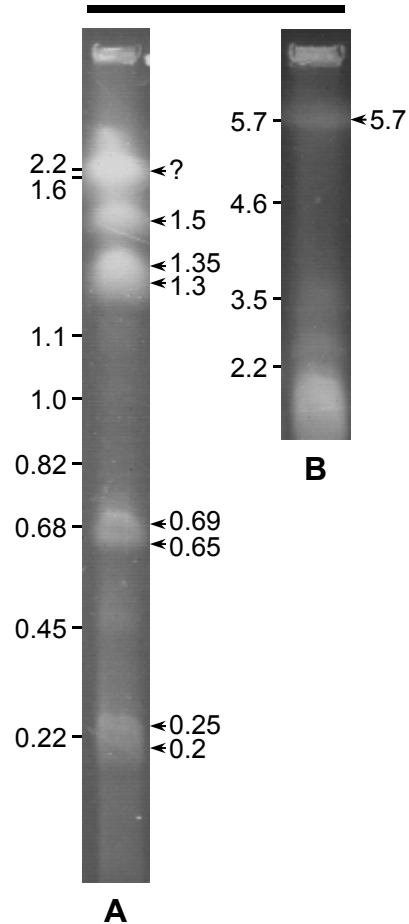

EU05, *PsiI*

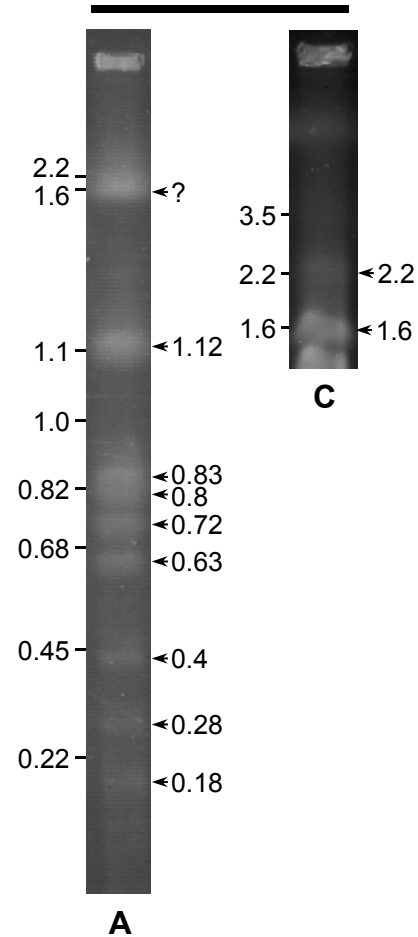

EUr01, *PsiI*

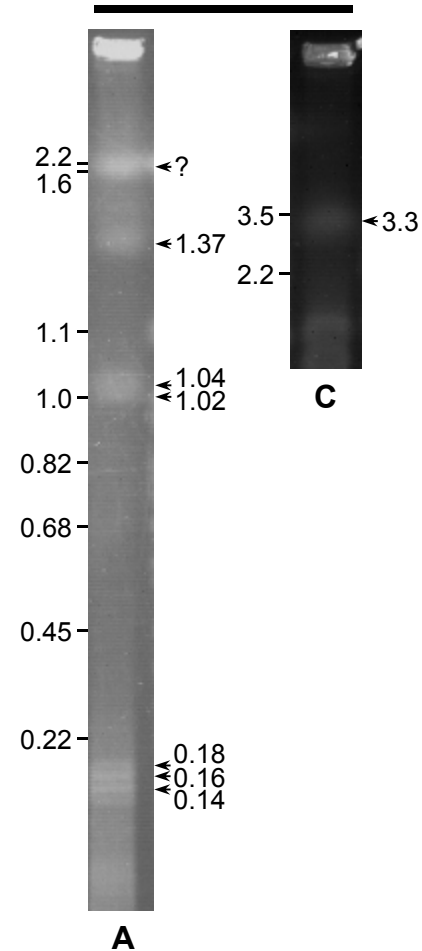

Supplement: Supplementary file 3 — Additional file 3: Electropherograms from PFGE. PDF file (.pdf) containing gel images from PFGE. Numbers on the left side of the image are Mbp of size standards (chromosomal DNA of S. cerevisiae and S. pombe). Numbers with arrowheads on the right side of images are fragment sizes estimated from the size standards. Conditions for electrophoresis are indicated under the image: (A) 0.5× TBE (45 mM Tris base, 45 mM borate, and 1 mM EDTA [pH 8.5]) with 10 mM thiourea, 1% agarose, 6 V cm-1 voltage, 60–120-s pulse time, 120° field angle, and 24-h run time at 13°C; (B) 1× TAE (40 mM Tris-acetate, 2 mM EDTA [pH 8.5]) with 10 mM thiourea, 0.8% agarose, 2 V cm-1 voltage, 1200–1800-s pulse time, 106° field angle, and 48-h run time at 14°C; (C) 1× TAE with 10 mM thiourea, 0.8% agarose, 3 V cm-1 voltage, 120–1200-s pulse time, 106° field angle, and 24-h run time at 13°C. (PDF 509 KB) [file 12864_2013_6303_MOESM3_ESM.pdf]
