# Supplementary material for: Different dynamics of genome content shuffling among host-specificity groups of the symbiotic actinobacterium Frankia
Source: BMC Genomics. 2014 Jul 19;15(1):609. doi: 10.1186/1471-2164-15-609 (PMC4117964; doi:10.1186/1471-2164-15-609)
Supplement: Supplementary file 2 — Additional file 2: Results of genomic PCR. PDF file (.pdf) containing electropherograms of PCR products. Genomic DNAs from Asi1, Ceq1, and Ema2 were used as templates. (PDF 208 KB) [file 12864_2013_6303_MOESM2_ESM.pdf]

Additional file 2

Asi1 (Alnus strain)

	Reference (kb)	Amplified (kb)
FRAAL5446	0.9	0.4*
FRAAL0105	0.9	0.9
FRAAL2465	1	2#
FRAAL4776	1.4	0.6*
FRAAL5831-FRAAL5832	0.5	2#
FRAAL6575-FRAAL6576	0.6	4#
FRAAL1975-FRAAL1976	0.7	0.6
FRAAL4400-FRAAL4401	1.2	0.7*
FRAAL1887 to IGR FRAAL1891-1892	6.2	2*
FRAAL5499 to IGR FRAAL5505-5506	6.4	1.6*
IGR FRAAL4786-4787 to IGR FRAAL4791-479	6.5	1.5*
IGR FRAAL0794-0795 to FRAAL0799	6.5	0.4*
IGR FRAAL1336-1337 to IGR FRAAL1339-1340	7.3	0.7*
IGR FRAAL3627-3628 to IGR FRAAL3637-3638	7.9	2.5*
FRAAL018 to FRAAL0194	12	2*
FRAAL6317 to FRAAL6382	80	2*

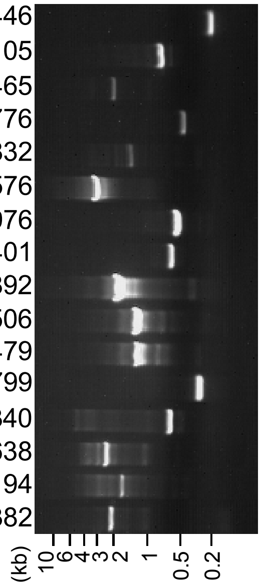

Ceq1 (Casuarina strain)

	Reference (kb)	Amplified (kb)
Francci3_4198	3.7	0.8*
Francci3_4067	2.2	0.7*
Francci3_2712	2.3	0.8*
Francci3_2708	2.3	0.8*
Francci3_1018 to Francci3_1019	2.2	0.7*
IGR Francci3_0309-0310 to IGR Francci3_0311-0312	3.5	0.9*
Francci3_3297 to IGR Francci3_3348-3349	72	1*
IGR Francci3_2571-2572 to Francci3_2579	17	1.5*
IGR Francci3_1704-1705 to Francci3_1763	56	2*
Francci3_1194 to IGR Francci3_1203-R0023	11	0.7*
IGR Francci3_2037-2038 to IGR Francci3_2055-2057	25	7*

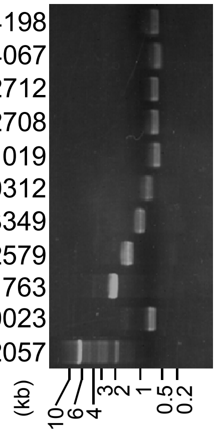

Ema2 (Elaeagnus strain)

	Reference (kb)	Amplified (kb)
Franean1_5590	1.6	0.4*
Franean1_3909	1.6	0.4*
Franean1_3354	1.8	0.9*
Franean1_3748	1.9	1.2*
Franean1_2409	2.3	0.6*
IGR Franean1_4748-4749	0.7	1#
IGR Franean1_5114-5116	1	2#
IGR Franean1_2198-2200	1.2	0.3*
IGR Franean1_3884-3886	1.4	1.5
IGR Franean1_1898-1900	2.2	0.5*
IGR Franean1_1307-1308 to Franean1_1312	5.4	1.5*
Franean1_3875 to IGR Franean1_3878-3879	6.6	6
Franean1_3483 to IGR Franean1_3489-3490	8.5	1.5*
Franean1_4792 to Franean1_4796	9.5	1*
Franean1_4246 to IGR Franean1_4253-4254	18	0.4*
IGR Franean1_5938-5939 to IGR Franean1_5948-5949	38	2.5*

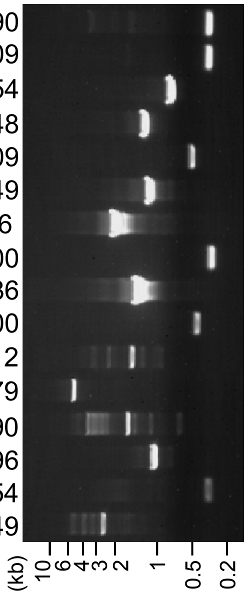

Strain	Asi1	Ceq1	Ema2
Reference > Amplified (*)	11 (69%)	11 (100%)	12 (75%)
Reference = Amplified	2 (12%)	0 (0%)	2 (12.5%)
Reference < Amplified (#)	3 (19%)	0 (0%)	2 (12.5%)
Total	16 (100%)	11 (100%)	16 (100%)
